# Genome-Wide Association Study of Body Conformation and Carcass Composition Traits in Landrace Pigs

**DOI:** 10.3390/ani16203316

**Published:** 2026-10-21

**Authors:** Shaoxiong Deng, Donglin Ruan, Zilun Pu, Zhibin Cao, Linhao Huang, Meng Lin, Jia-Jin Wu, Enqin Zheng, Jie Yang, Langqing Liu, Hui Zhang

**Affiliations:** 1 State Key Laboratory of Swine and Poultry Breeding Industry, College of Animal Science and National Engineering Research Center for Breeding Swine Industry, South China Agricultural University, Guangzhou 510642, China; 2 National and Regional Livestock Genebank, Guangdong Gene Bank of Livestock and Poultry, South China Agricultural University, Guangzhou 510642, China; 3 Guangdong Provincial Key Laboratory of Agro-Animal Genomics and Molecular Breeding, South China Agricultural University, Guangzhou 510642, China; 4 School of Life Science, Anhui Medical University, Hefei 230032, China

**Keywords:** GWAS, Landrace pig, body conformation, carcass composition

## Abstract

Body conformation and carcass composition are economically important traits in pig production, yet their underlying genetic mechanisms remain incompletely characterized. To dissect the genetic bases of body length (BL), body height (BH), backfat thickness (BFT), and lean meat percentage (LMP), we estimated genetic parameters and performed a genome-wide association study (GWAS) using 50 K SNP array genotypes from 1527 Landrace pigs. Phenotypic variance decomposition revealed moderate-to-high heritability for all four traits, ranging from 0.23 for BH to 0.43 for BL. Genetic correlation analysis uncovered a strong negative correlation (−0.80 ± 0.05) between BFT and LMP, as well as a moderate positive correlation (0.46 ± 0.03) between BL and BH. Our GWAS identified 14 SNP–trait associations involving 10 unique SNPs and two genomic regions with overlapping association signals. On *Sus scrofa* chromosome 1 (SSC1), overlapping signals for BFT and LMP occurred near *ABL1* and *QRFP*, whereas signals for body conformation mapped to SSC17 near *BMP2*, *PROKR2*, *SLC23A2*, and *PCNA*. These genes are positional and biologically plausible candidates, and these candidate markers hold potential for genomic selection to simultaneously improve body size and meat-production performance.

## Simple Summary

Improving pork production efficiency is a primary goal for the agricultural industry. While traits like body size (length and height), backfat thickness (BFT), and lean meat percentage (LMP) directly determine meat yield, their genetic bases remains incompletely understood. This study investigated the genetic architecture of these traits in Landrace pigs. We identified 10 unique genetic markers (SNPs) and highlighted two shared genomic regions: one influencing both BFT and LMP, and another affecting body length and height. These findings advance our understanding of pig growth biology. These candidate markers hold potential for genomic selection to simultaneously improve body size and meat yield.

## 1. Introduction

Pork is a major source of animal protein worldwide, and improving production efficiency remains a central objective of pig breeding [1,2]. In commercial pig production, body conformation traits (e.g., body length, BL; body height, BH) and carcass composition traits (e.g., backfat thickness, BFT; lean meat percentage, LMP) represent critical economic indicators. BL and BH reflect skeletal development and body capacity, directly determining animal body framework and growth potential. A longer body frame supports heavier slaughter weights and increases carcass length, which is genetically correlated with higher yields of high-value primal cuts and overall meat output [3]. Meanwhile, BFT and LMP describe adipose-muscle tissue distribution, which strongly governs carcass market value, feed conversion efficiency, and consumer acceptance of pork products. Backfat thickness is a key indicator of subcutaneous fat deposition; excessive backfat increases feed cost and produces low-value fat tissue, while insufficient fat deposition may compromise meat juiciness and flavor [4]. Lean meat percentage quantifies the proportion of edible lean muscle tissue of the carcass and is one of the primary breeding targets for modern pig lines. Genetically, BFT and LMP are tightly interrelated: selection for reduced backfat generally elevates lean meat yield, yet intense selection for a high lean proportion sometimes leads to undesirable side-effects such as deteriorated meat quality. Balancing backfat deposition and lean muscle accretion is therefore essential to satisfy both processor economic demands and consumer sensory preferences for pork [5].

Previous genetic evaluations have consistently demonstrated that body conformation and carcass traits in pigs possess moderate-to-high heritability, rendering them highly responsive to genetic selection [3,6,7,8,9]. Genome-wide association studies (GWAS) are widely used to dissect the genetic architecture of complex quantitative traits in pigs.Hundreds of quantitative trait loci (QTLs) related to body conformation and carcass composition traits have been accumulated in the Pig QTLdb database (https://www.animalgenome.org/cgi-bin/QTLdb/SS/index, accessed on 21 August 2026). Among them, 173 and 632 QTLs are associated with body height (BH) and body length (BL), respectively. Additionally, 2858 QTLs for subcutaneous fat thickness and 185 QTLs for lean meat percentage have been documented in this public resource [10].

Within these identified QTL regions, several major functional genes have been successfully pinpointed. For body conformation, notable examples include the *VRTN* gene, which governs the number of vertebrae and directly impacts carcass length [11,12,13], and the *BMP2* gene, which has been significantly associated with both body length and height in large pig populations [12,14]. Regarding carcass composition, several major candidate genes—such as *MC4R* and *PPARD*—have been associated with backfat thickness, fat deposition, and lean meat yield across multiple commercial breeds [9,15].

Despite these reported loci, the specific causal mutations and mechanisms linking body conformation and carcass composition remain to be fully elucidated. Therefore, this study aimed to characterize the genetic architecture of body conformation (BL, BH) and carcass composition (BFT, LMP) traits in a Landrace pig population. We conducted a genome-wide association study (GWAS) to identify significant SNP–trait associations and candidate genomic regions, and used genomic restricted maximum likelihood (GREML) analyses to estimate trait heritabilities and genetic correlations. Our results identify population-specific association signals and overlapping genomic regions for BFT and LMP and for BL and BH. These overlaps may reflect shared biological regulation or linkage disequilibrium between distinct causal variants. These results provide novel molecular insights and promising genetic markers to support genomic selection for improved body conformation and meat-production efficiency in pig-breeding programs.

## 2. Materials and Methods

### 2.1. Animals and Phenotypic Data

In this study, 1527 Landrace pigs from one core breeding farm of Jiangxi Jiada Group Co., Ltd. (Ganzhou, China) were included. Phenotypes were recorded in 2023 at the end of routine performance testing. Phenotypic measurements were recorded when the pigs reached a target body weight of 115 ± 5 kg, corresponding to an approximate age of 150 to 180 days and the male-to-female ratio was approximately 1:2. Body height was measured as the vertical distance from the highest point of the shoulder to the ground using a measuring stick. Body length was measured along the body contour from the midpoint of the ear to the base of the tail using a flexible tape measure. Carcass-composition traits were measured in vivo using an Aloka SSD-500 B-mode ultrasound scanner (Aloka Co., Ltd., Tokyo, Japan). Backfat thickness (BFT) was measured at the 3rd–4th last intercostal space, and loin muscle area (LMA) was measured during the same examination. The observed measurements were adjusted to a standard body weight of 115 kg. For non-boars, adjusted BFT was calculated as BFT115 = BFTobs + (115 − Wobs) × [BFTobs/(Wobs − 2.268)], whereas for boars it was calculated as BFT115 = BFTobs + (115 − Wobs) × [BFTobs/(Wobs + 9.072)]. Observed LMA was adjusted as LMA115 = LMAobs + (115 − Wobs) × [LMAobs/(Wobs + 60.307)]. Here, BFTobs and LMAobs denote measurements at the end of the performance test, and Wobs denotes body weight at measurement. LMP was not obtained by scaling an observed LMP value; it was re-estimated from adjusted BFT and LMA using LMP115 = (80.95 − 0.6472 × BFT115 + 0.7274 × LMA115) × 0.54. BFT and LMP values reported in the summary statistics and genetic analyses were adjusted values, whereas BL and BH were recorded phenotypes. Because all animals were measured in one farm and one recording year, the findings primarily describe this management and genetic population and should not be assumed to represent all Chinese Landrace pigs.

### 2.2. Genotypes and Quality Control

Genomic DNA was extracted from ear tissue using a standard phenol/chloroform method and diluted to 50 ng/μL. The 1527 Landrace pigs were genotyped using the Zhongxin I 50 K chip (Beijing Compass Biotechnology Co., Ltd., Beijing, China). Genotype QC was conducted using PLINK v1.90 software [16]. Individuals with a missing genotype rate > 5% were removed. SNPs with a call rate < 0.90 or MAF < 0.05 were excluded. Duplicate samples, and sex or sample identity inconsistencies were checked during sample curation; no additional samples were removed after these checks. Only SNPs on the 18 autosomes were retained, and SNPs on sex chromosomes or unmapped regions were excluded. After QC, 1527 pigs and 36,706 SNPs were available for analysis.

### 2.3. Estimation of Genetic Parameters

We estimated SNP-based heritability for each trait and genetic correlations between traits using GREML in GCTA (GCTA v1.94.0 beta) [17]. The univariate model was

*y* = *Wα* + *Zu* + *e*

where age, sex, and the first five principal components were included as fixed effects or covariates, consistent with the GWAS model. Here, *y* is the vector of phenotypic values; *W* is the incidence matrix for fixed effects and covariates; *α* is the corresponding coefficient vector; *Z* relates records to animals; *u* is the random additive genetic effect, assumed to follow *u* ~ *N*(0,*Kσ^2^u*), where *K* is the additive genomic relationship matrix; and *e* is the residual vector, assumed to follow *e* ~ *N*(0, *Iσ^2^e*). The same additive GRM was used to account for genomic relatedness in all GREML analyses. GCTA REML analyses were accepted when the program returned a valid converged solution. Bivariate GREML used the same fixed effects and covariates for both traits.

Furthermore, to assess the genetic correlation between two traits, we conducted a bivariate GREML analysis using GCTA. The genetic correlation coefficient (*r_g_* ) was calculated using the following formula:

$$r_{g\;}=\frac{{\mathrm{cov}}_{g12}}{\sqrt{\sigma_{g1}^{2}\sigma_{g2}^{2}}}$$

where *r_g_* is the genetic correlation between two traits, *cov_g_*_12_ is their additive genetic covariance, and $\sigma_{g1}^{2}$ and $\sigma_{g2}^{2}$ are the corresponding additive genetic variances.

### 2.4. Phenotypic Variation Explained

*PVE* (Phenotypic variation explained) by a given SNP was calculated using the following formula [18]:

$$\mathrm{PVE}=\frac{2\beta^{2}\mathrm{MAF}(1-\mathrm{MAF})}{2\beta^{2}\mathrm{MAF}(1-\mathrm{MAF})+\mathrm{SE}(\beta {)}^{2}\times 2\mathrm{NMAF}(1-\mathrm{MAF})}$$

*PVE* values were calculated for each SNP using effect size estimates (*β*), standard errors (*SE*), *MAF*, and the sample size (*N*) derived from GWAS summary statistics. *β* represents the effect size of the SNP obtained from the GWAS. *MAF* denotes the minor allele frequency of the SNP. *SE* refers to the standard error of the effect size. *N* is the sample size for the SNP in the trait.

### 2.5. Association Analyses

To account for population stratification, PCA was performed on all retained SNPs using GCTA v1.94.0 beta [17]. The first five principal components were included as covariates in both the GWAS and GREML models. Five PCs were retained because they captured the major population structure in this population and reduced the genomic inflation factor (λ) to approximately 1.0; additional PCs did not further improve λ.

The genome-wide association analysis was conducted using the GCTA MLMA mixed linear model. Age, sex, and PCs 1–5 were included as fixed effects or covariates, and the additive genomic relationship matrix was used to model the polygenic background. The model was

*y* = *Wα* + *Xβ* + *Zu* + *e*

Here, *y* is the vector of phenotypic observations for each trait; *W* is the incidence matrix of fixed effects and covariates, including the overall mean, sex, age at recording, and the first five principal components (PCs 1–5) estimated from the genomic relationship matrix (GRM) to account for population structure; *α* is the corresponding vector of fixed-effect coefficients; *X* is the vector of SNP genotypes (coded as 0, 1, or 2 copies of the reference allele) for the tested marker; *β* is its allele-substitution effect, fitted as a fixed effect; *Z* is the incidence matrix relating phenotypic records to animals; *u* is the vector of random polygenic effects with *u* ~ *N*(0,*Kσ^2^u*), where *K* is the additive genomic relationship matrix computed from all SNPs and *σ^2^u* is the polygenic genetic variance; and *e* is the vector of residual errors with *e* ~ *N*(0, *Iσ^2^e*) where *I* is the identity matrix and *σ^2^e* is the residual variance. The genome-wide threshold was *p* < 0.05/36,706 (approximately 1.36 × 10^−6^), and the suggestive threshold was *p* < 1/36,706 (approximately 2.72 × 10^−5^) [19]. The genomic inflation factor λ was calculated as the ratio of the median observed chi-square statistic to the median expected chi-square statistic from a chi-square distribution with 1 degree of freedom under the null hypothesis.

Candidate genes were screened within 500 kb upstream and downstream of each associated SNP, based on the observed LD-decay distance at r^2^ = 0.2. Gene annotation was based on the *Sus scrofa* 11.1 reference genome and the Ensembl version Sscrofa11.1.111 [20]. Gene proximity and biological plausibility were used to prioritize the candidate genes.

## 3. Results and Discussion

### 3.1. Phenotype Statistics and Genetic Correlation Among the Traits

Table 1 presents the summary statistics for the analyzed traits in the Landrace pig. The population demonstrated considerable phenotypic diversity across both body conformation and carcass composition metrics. Specifically, the average adjusted backfat thickness (BFT) and estimated lean meat percentage (LMP) were 14.44 mm and 54.91%, respectively. For body conformation traits, the mean body length (BL) was 104.15 cm and mean body height (BH) was 63.98 cm. Phenotypic ranges were wide, with BFT from 7.17 mm to 28.71 mm and BL from 87 cm to 123 cm. The coefficients of variation (CV) revealed different levels of sample variability among the traits. BFT displayed the highest phenotypic variability with a CV of 20.69%, suggesting substantial room for genetic selection and modification of fat deposition. Conversely, LMP, BL, and BH exhibited relatively lower CV values of 4.38%, 5.10%, and 3.46%, respectively.

The SNP-based heritability estimates ranged from 0.23 ± 0.04 for BH to 0.43 ± 0.05 for BL (Table 1), indicating moderate-to-high additive genetic contributions. These estimates demonstrate that genetic factors make major contributions to phenotypic variation, supporting the feasibility of genomic selection to accelerate genetic improvement for these traits.

**Figure 1:**
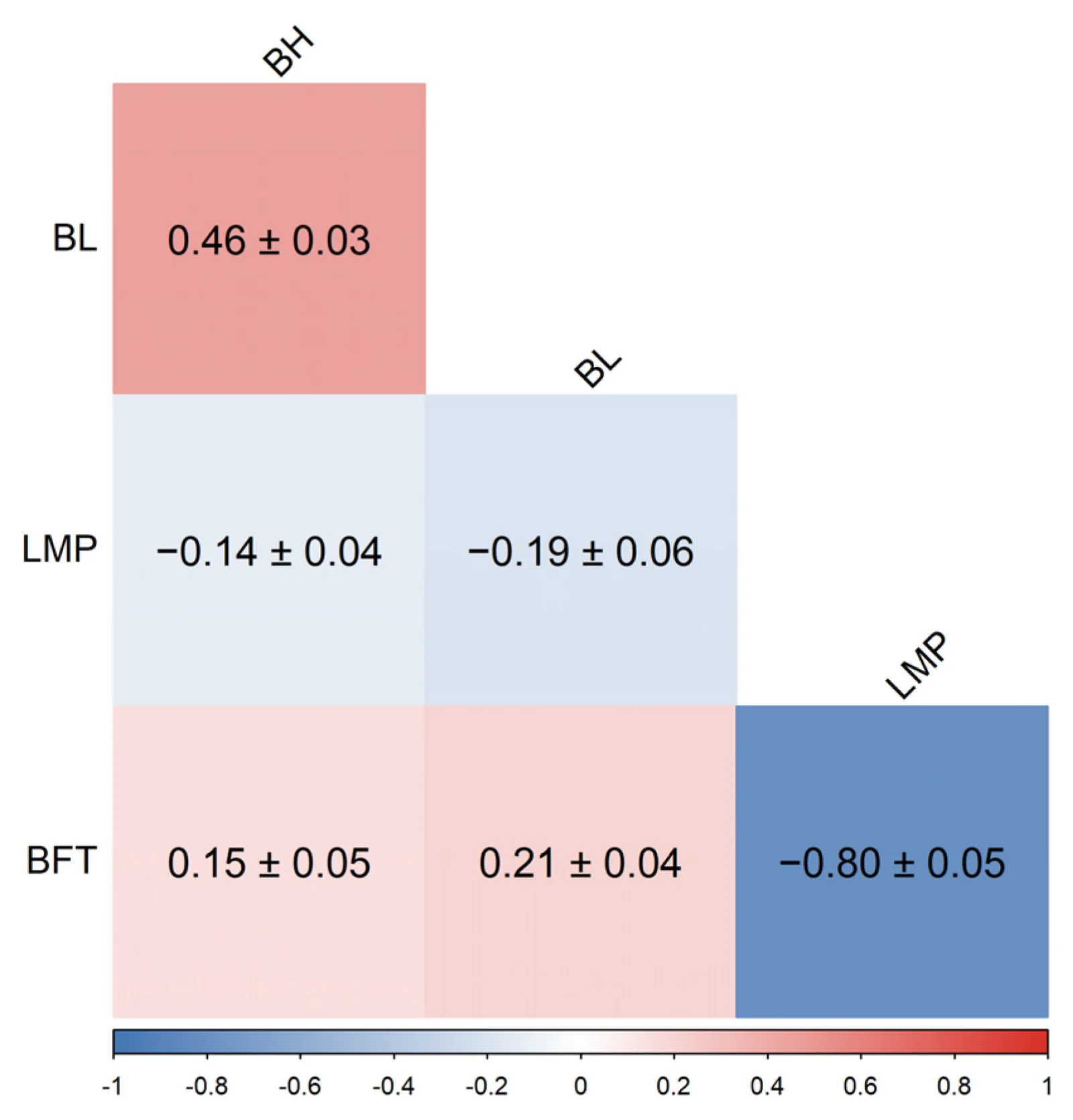
Genetic correlations between the BL, BH, LMP, and BFT traits in the Landrace pig populations.

Estimation of genetic correlations is essential to guide multi-trait selection schemes in pig breeding. We detected a strong negative genetic correlation between LMP and BFT (*r_g_* = −0.80 ± 0.05) (Figure 1). This estimate is compatible with a biological trade-off between adipose deposition and muscle accretion, potentially involving insulin/IGF signalling and the balance between adipogenesis and myogenesis [21]. However, because LMP was re-estimated from ultrasound variables that include BFT, part of the observed correlation may arise from the mathematical construction of LMP rather than independent biological pleiotropy.

Regarding body conformation traits, BL and BH demonstrated a moderate positive genetic correlation (*r_g_* = 0.46 ± 0.03) (Figure 1), suggesting that skeletal growth along the longitudinal and vertical axes is genetically synchronized. Consequently, selection for increased body length will generally yield taller individuals. Our estimated correlation falls within the range of values reported in recent studies. A higher genetic correlation of 0.63 was previously documented in a purebred Large White population [12], whereas a lower estimate of 0.20 was observed in a three-way crossbred commercial population [22]. This variation in magnitude likely reflects differences in population-specific genetic architectures, allele frequencies, and selection histories across diverse breeds. Nevertheless, the consistently positive genetic correlations observed across these distinct swine populations further substantiate the existence of a shared genetic basis governing overall skeletal elongation. Interestingly, both BL and BH exhibited weak positive genetic correlations with BFT (0.21 ± 0.04 and 0.15 ± 0.05, respectively) and weak negative genetic correlations with LMP (−0.19 ± 0.06 and −0.14 ± 0.04, respectively). These low-magnitude estimates are consistent with previous quantitative genetic studies. For instance, Johnson and Nugent [23] reported a comparably weak positive genetic correlation (0.11) between body length and backfat thickness in swine. From a breeding perspective, exclusive selection for larger body size may slightly increase subcutaneous fat deposition and suppress lean-muscle gain. Accordingly, balanced breeding objectives and optimized selection indices are required to jointly improve body conformation and carcass performance.

### 3.2. Genome-Wide Association Studies

**Figure 2:**
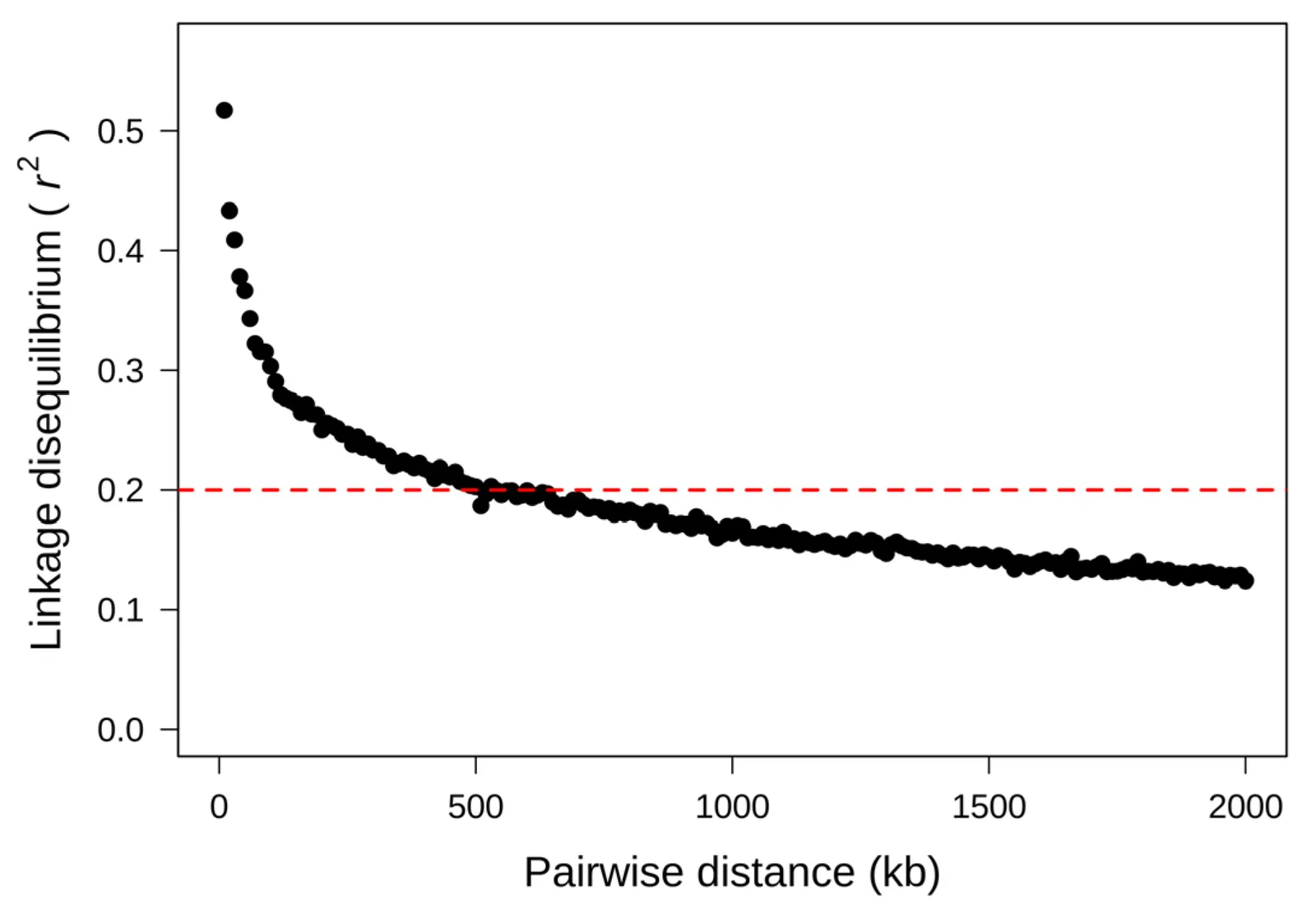
Linkage disequilibrium decay across the whole genome of the association panel. The red line represents the linkage disequilibrium threshold for the association panel (*r^2^* = 0.2).

After QC, 36,706 SNPs with genotypes on 1527 Landrace pigs were retained for GWAS analysis. The genome-wide significance threshold was set at *p* < 0.05/36,706 (approximately 1.36 × 10^−6^), and the suggestive significance threshold was set at *p* < 1/36,706 (approximately 2.72 × 10^−5^). The GWAS λ ranged from 0.91 to 1.00, indicating minimal population inflation in GWAS. In this study, the linkage disequilibrium (LD) decay analysis revealed that the average *r^2^* dropped to a threshold of 0.2 at a physical distance of approximately 500 kb (Figure 2). This observed LD decay pattern in our Landrace population is consistent with previous estimates reported for Western commercial Landrace pigs by Ai et al. [24].

**Figure 3:**
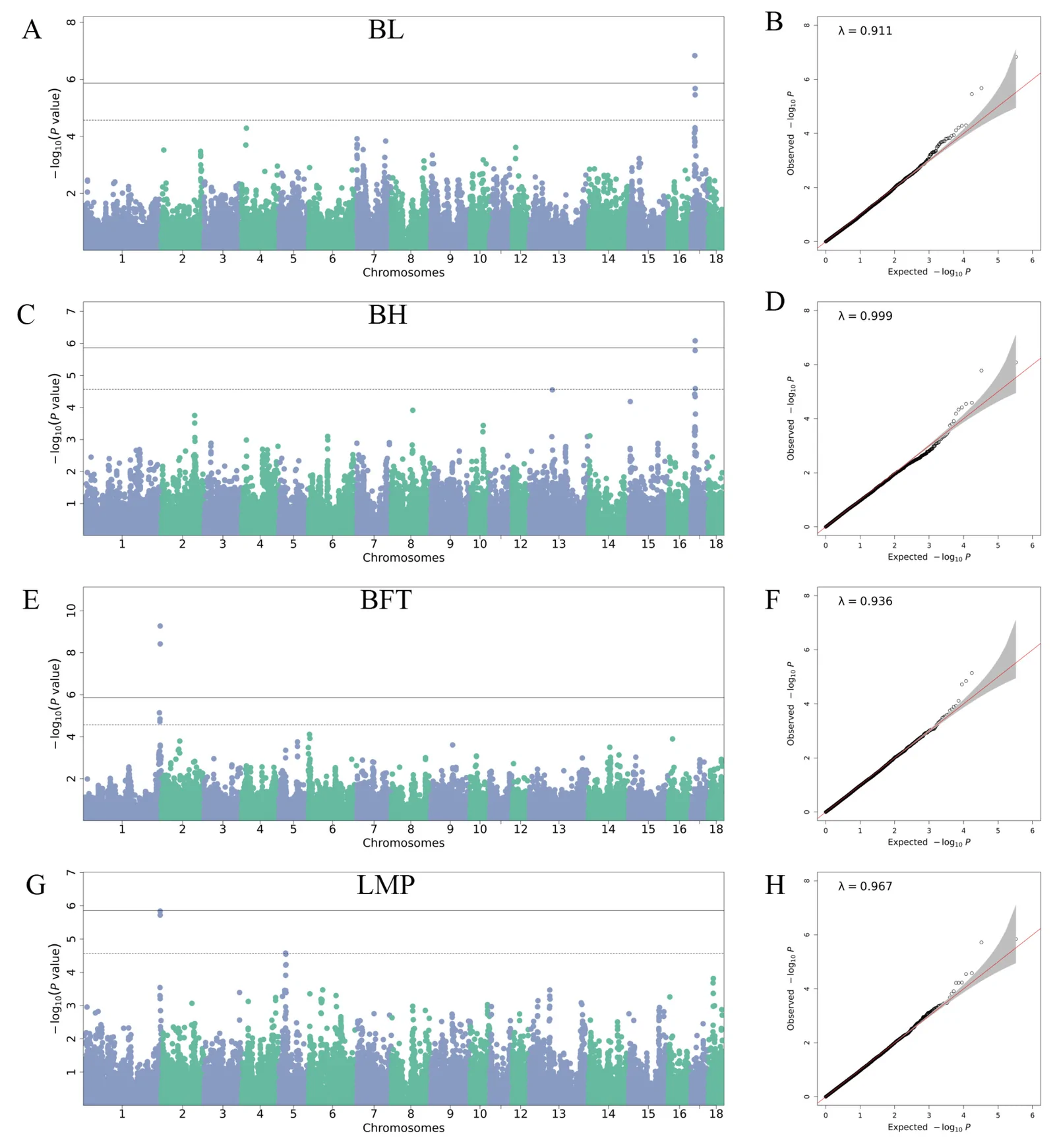
Manhattan and Q-Q plots from GWAS for BL (**A**,**B**), BH (**C**,**D**), BFT (**E**,**F**), and LMP (**G**,**H**). Solid horizontal lines represent genome-wide significance thresholds; dashed lines denote suggestive significance thresholds. Genomic inflation factor λ is labelled on each Q-Q plot.

GWAS results are summarized in Table 2, and the corresponding Manhattan and quantile-quantile (Q-Q) plots are presented in Figure 3A–H. A total of 14 SNP–trait associations involving 10 unique SNPs were identified, including four genome-wide significant and ten suggestive associations. For carcass composition traits, we identified five SNPs located on *Sus scrofa* chromosome (SSC) 1 associated with BFT, comprising two genome-wide significant and three suggestive SNPs. Additionally, three suggestive SNPs distributed across SSC 1 and SSC 5 were found to be associated with the LMP. Notably, the leading SNP for BFT on SSC 1 explained up to 2.46% of the phenotypic variance. For body conformation traits, all significant signals were mapped to SSC 17. We identified three SNPs associated with body height (one genome-wide significant and two suggestive) and three SNPs associated with body length (one genome-wide significant and two suggestive), which explained 1.35% to 2.09% of the phenotypic variance.

**Figure 4:**
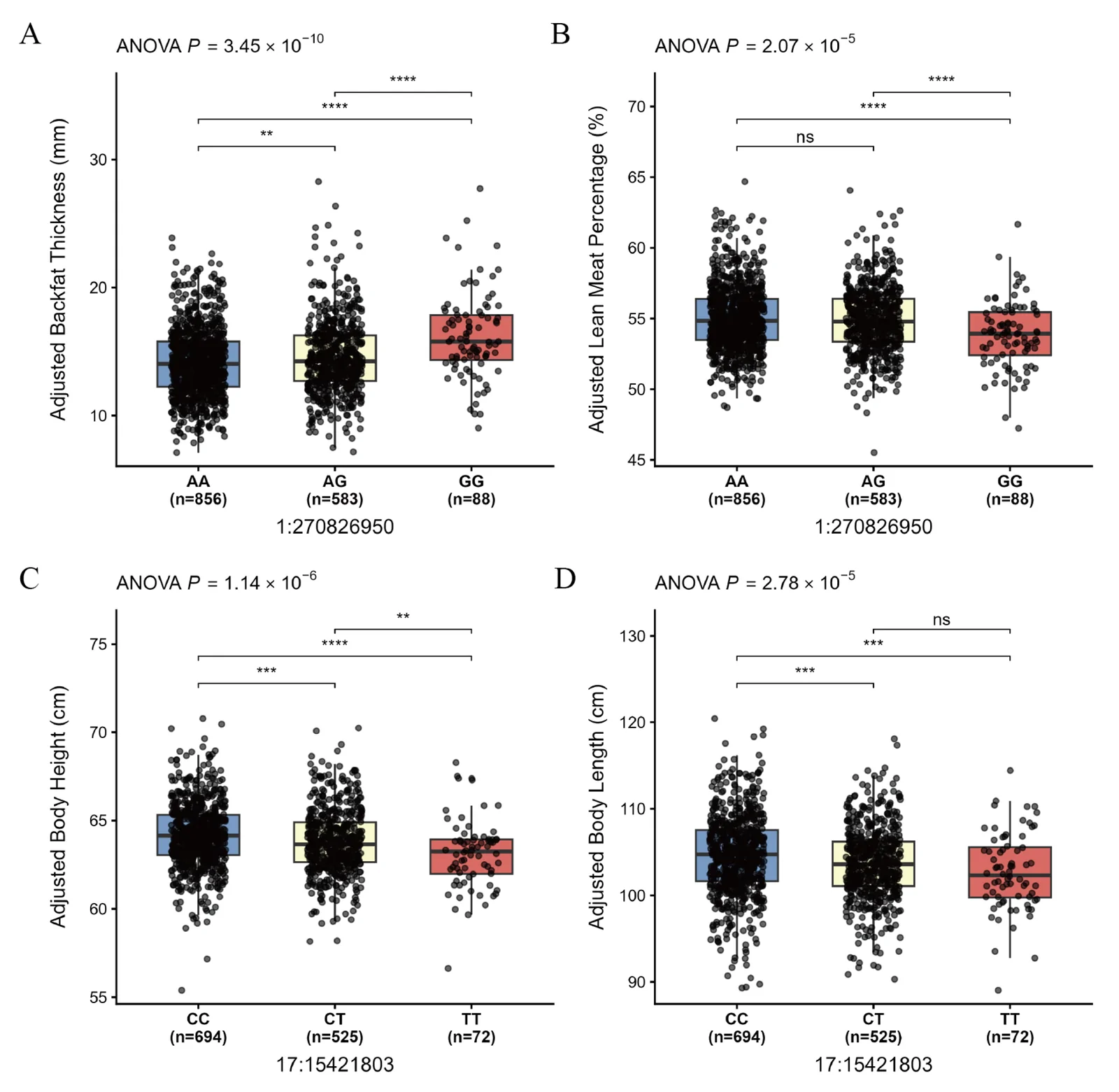
Distribution of adjusted phenotypes across genotypes of peak SNPs identified in the GWAS. Box plots show (**A**) adjusted backfat thickness and (**B**) adjusted lean meat percentage across genotypes of SNP 1:270826950; (**C**) adjusted body height and (**D**) adjusted body length across genotypes of SNP 17:15421803. Overall differences among genotypes were assessed by one-way ANOVA (*p* values shown in each panel), and pairwise comparisons were performed using Welch’s *t*-test (ns, not significant; ** *p* < 0.01; *** *p* < 0.001; **** *p* < 0.0001).

Consistent with their strong negative genetic correlation, BFT and LMP shared overlapping association peaks on SSC1. The top shared marker CNC10015492 lies within the intron of *ABL1*. Adjusted genotype–phenotype analysis at this locus (Figure 4A,B) revealed that individuals carrying the favorable AA genotype exhibited 2.06 mm lower BFT and 1.21% higher LMP, compared with those with the GG genotype. *ABL1* encodes a non-receptor tyrosine kinase that shuttles between the nucleus, cytoplasm, and nucleolus and participates in cell division, adhesion, differentiation, and stress responses. Recent genomic studies on commercial pig populations have proposed *ABL1* as a promising candidate gene located in structural-variation hotspots potentially associated with backfat thickness and subcutaneous fat deposition [25]. Furthermore, multiple independent studies have corroborated its extensive pleiotropic effects on growth and carcass traits. For instance, Huang et al. [26] reported significant associations between this gene and both body weight and BFT, while other studies have linked it to average daily gain (ADG) and age to 105 kg [27]. Additionally, *ABL1* has been explicitly identified as a crucial regulator of fat metabolism in Yorkshire pigs [28]. Close to this peak, several other functionally related genes were identified. For instance, the *QRFP* gene, located 84.93 kb away from the locus CNC10015492, encodes a pre-proprotein that yields multiple mature protein products belonging to the RFamide neuropeptide family. These peptides can activate the G-protein-coupled receptor, which drives orexigenic effects in rodents [29,30]. Beyond stimulating food intake, *QRFP* promotes fat mass gain, increases body weight and adipogenesis, and suppresses thermogenesis [29,31,32]. The overlapping GWAS signals for both BFT and LMP within this genomic window on SSC1 suggest that this region may contribute to the genetic basis of the strong negative genetic correlation observed between these two traits, although the underlying mechanism remains to be functionally validated. This shared region may also be potentially involved in the allocation of nutritional resources between lean muscle accretion and subcutaneous fat deposition, which warrants further functional investigation.

All association signals for body conformation traits mapped to SSC17. Overlapping peaks for BH and BL were observed for markers such as CNC10170313 and CNC10170312. Genotype–phenotype analysis for CNC10170313 (Figure 4C,D) showed that animals carrying the CC genotype exhibited 1.06 cm greater BH and 1.98 cm greater BL compared with TT-homozygous individuals. These phenotypic observations corroborate the moderate positive genetic correlation (0.46 ± 0.03) observed between these two traits, a relationship similarly documented in other commercial swine populations [12]. These overlapping association signals on SSC17 suggest that this genomic region may potentially affect both the longitudinal (BL) and vertical (BH) expansion of the body framework in this Landrace population; however, formal colocalization or fine-mapping analyses are required to confirm a shared causal variant.

Within these candidate regions, *BMP2* (Bone morphogenetic protein 2) represents one of the most promising biological candidate genes. It encodes a potent osteogenic factor exerting fundamental functions in bone and cartilage development, osteoblast differentiation, and overall skeletal patterning [33,34]. Multiple pig GWAS have detected body-size associated genomic signals residing adjacent to *BMP2*. Li et al. (2021) characterized a functional regulatory mutation upstream of *BMP2* affecting carcass length in DLY crossbred pigs [35]. Among published GWAS for in-vivo body conformation traits, co-localized association peaks for both body length and body height were observed in one Large White population [12].

Furthermore, the leading genome-wide significant SNP for body length (CNCB10011715) was located directly within the *PROKR2* gene. While known for its role in neuroendocrine regulation, the surrounding genomic window also encompasses highly relevant structural and proliferative genes. For instance, *SLC23A2* (−240.31 Kb) encodes a vitamin C transporter that is essential for collagen synthesis and the structural integrity of the bone extracellular matrix. Additionally, *PCNA*, located 109.27 Kb upstream of the leading genome-wide significant SNP (CNCB10011715) on SSC 17, was identified as a strong candidate gene for body length. As a universal master regulator of cellular proliferation, *PCNA* serves as a definitive marker for active chondrocyte proliferation within the growth plate [36]. This robust chondrocyte proliferation provides the essential cellular basis for the longitudinal growth of metapodials and long bones. Therefore, we hypothesize that genetic variations near *PCNA* may regulate body length in pigs by modulating the proliferation rate of chondrocytes during skeletal elongation. Together, this gene cluster on SSC 17 highlights multiple promising candidate genes involved in the regulation of skeletal dimensions. Further validation is therefore needed for their roles in swine breeding programs. An important limitation of the present study is that the candidate genes were prioritized primarily according to their genomic proximity to the associated variants and previously reported biological functions. Although these criteria provide useful biological interpretation and facilitate the prioritization of candidate genes, they do not establish a causal relationship between the associated variants and the proposed genes. Tissue-specific expression analysis, eQTL investigation, and functional experiments will therefore be required to determine the causal genes and elucidate the underlying molecular mechanisms of the identified associations.

## 4. Conclusions

In this study, we conducted a GWAS for body length (BL), body height (BH), backfat thickness (BFT), and lean meat percentage (LMP) using 1527 Landrace pigs. All four traits displayed moderate-to-high SNP-based heritability, ranging from 0.23 to 0.43. A strong negative genetic correlation (−0.80 ± 0.05) was observed between BFT and LMP, whereas BL and BH showed a moderate positive genetic correlation (0.46 ± 0.03). We detected 14 SNP–trait associations involving 10 unique SNPs and identified two candidate shared genomic regions. One shared region on SSC 1, which contains *ABL1* and *QRFP*, was associated with the variation in backfat thickness and lean meat percentage. The SSC 17 region harboring *BMP2*, *PROKR2*, *SLC23A2*, and *PCNA* potentially influences both body length and body height. These loci represent promising candidate markers that require validation in independent Landrace populations, as well as functional characterization, before their utility in genomic or marker-assisted selection can be established.

## 5. Patents

One patent application related to this work has been filed with the National Intellectual Property Administration (NIPA) of the People’s Republic of China with South China Agricultural University as the applicant: “Application of an SNP Molecular Marker Associated with Body Length and Body Height on Pig Chromosome 17” (application no. 202611561065.2, filed on 10 October 2026). The patent application is currently pending and has not yet been granted.

## Figures and Tables

**Table 1:** Summary statistics of body conformation and carcass composition traits in Landrace pigs.

Trait ^1^	Mean ± SD ^2^	Min ^3^	Max ^4^	CV.% ^5^	*h*^2^ ± SE ^6^
BFT	14.44 ± 2.99	7.17	28.71	20.69	0.37 ± 0.04
LMP	54.91 ± 2.41	45.63	64.64	4.38	0.34 ± 0.04
BL	104.15 ± 5.31	87	123	5.1	0.43 ± 0.05
BH	63.98 ± 2.21	56	74	3.46	0.23 ± 0.04

^1^ Four body conformation and carcass composition Traits. ^2^ Mean ± standard deviation. ^3^ Minimum (Min). ^4^ Maximum (Max). ^5^ Coefficient of variation. ^6^ Heritability ± standard error.

**Table 2:** SNP–trait associations reaching genome-wide or suggestive thresholds for body conformation and carcass composition traits in Landrace pigs.

Trait	SSC ^1^	SNP ID ^2^	Position (bp)	MAF	Beta	SE	*p*-Value	Nearest Gene (Distance)	PVE (%) ^3^
BFT	1	CNC10015492	270,826,950	0.25	0.89	0.14	5.32 × 10^−10^	ABL1 (within)	2.46
	1	CNC10015502	271,019,295	0.31	0.78	0.13	3.80 × 10^−9^	LAMC3 (within)	2.22
	1	CNC10015438	268,278,308	0.22	0.67	0.15	7.23 × 10^−6^	CDK9 (within)	1.30
	1	CNCB10001491	270,354,493	0.34	0.55	0.13	1.43 × 10^−5^	NCS1 (−19.59 Kb)	1.22
	1	CNCB10001489	270,002,492	0.33	0.54	0.13	1.90 × 10^−5^	HMCN2 (+9.48 Kb)	1.18
LMP	1	CNC10015492	270,826,950	0.25	−0.56	0.12	1.44 × 10^−6^	ABL1 (within)	1.50
	1	CNC10015502	271,019,295	0.31	−0.51	0.11	1.90 × 10^−6^	LAMC3 (within)	1.46
	5	CNCB10003844	24,170,214	0.33	0.52	0.12	2.63 × 10^−5^	ENSSSCG00000050485 (−3.2 Kb)	1.14
BH	17	CNC10170313	15,421,803	0.26	−0.49	0.10	8.26 × 10^−7^	ENSSSCG00000043546 (−89.36 Kb)	1.85
	17	CNC10170312	15,401,211	0.27	−0.48	0.10	1.66 × 10^−6^	ENSSSCG00000043546 (−68.77 Kb)	1.75
	17	CNC10170325	15,896,846	0.20	−0.49	0.12	2.57 × 10^−5^	ENSSSCG00000044805 (within)	1.35
BL	17	CNCB10011715	14,252,013	0.31	−1.24	0.24	1.47 × 10^−7^	PROKR2 (within)	2.10
	17	CNC10170313	15,421,803	0.26	−1.19	0.25	2.09 × 10^−6^	ENSSSCG00000043546 (−89.36 Kb)	1.71
	17	CNC10170312	15,401,211	0.27	−1.17	0.25	3.50 × 10^−6^	ENSSSCG00000043546 (−68.77 Kb)	1.64

^1^ *Sus scrofa* chromosome. ^2^ Underlined SNP IDs denote genome-wide significant associations (*p* < 0.05/36,706); remaining associations meet the suggestive threshold (*p* < 1/36,706) ^3^ Phenotypic variation explained by each SNP.

## Data Availability

The data that support the findings of this study are available from the corresponding author upon reasonable request.

## Abbreviations

The following abbreviations are used in this manuscript:

GWAS	Genome-Wide Association Study
SNP	Single-Nucleotide Polymorphism
QTL	Quantitative Trait Locus
BL	Body Length
BH	Body Height
BFT	Backfat Thickness
LMP	Lean Meat Percentage
GREML	Genomic Restricted Maximum Likelihood
MLM	Mixed Linear Model
PCA	Principal Component Analysis
LD	Linkage Disequilibrium
CV	Coefficient of Variation
MAF	Minor Allele Frequency
SSC	*Sus scrofa* Chromosome
